# Enantiotropy of Simvastatin as a Result of Weakened Interactions in the Crystal Lattice: Entropy-Driven Double Transitions and the Transient Modulated Phase as Seen by Solid-State NMR Spectroscopy

**DOI:** 10.3390/molecules27030679

**Published:** 2022-01-20

**Authors:** Jiri Brus, Jiri Czernek, Martina Urbanova, Ctirad Červinka

**Affiliations:** 1 Department of NMR Spectroscopy, Institute of Macromolecular Chemistry, Czech Academy of Sciences, Heyrovsky Sq. 2, 162 06 Prague, Czech Republic; czernek@imc.cas.cz (J.C.); urbanova@imc.cas.cz (M.U.); 2 Department of Physical Chemistry, University of Chemistry and Technology Prague, Technická 5, 166 28 Prague, Czech Republic; Ctirad.Cervinka@vscht.cz

**Keywords:** polymorphism, enantiotropy, transient modulated phase, dynamics, entropy, solid-state nuclear magnetic resonance (NMR)

## Abstract

In crystalline molecular solids, in the absence of strong intermolecular interactions, entropy-driven processes play a key role in the formation of dynamically modulated transient phases. Specifically, in crystalline simvastatin, the observed fully reversible enantiotropic behavior is associated with multiple order–disorder transitions: upon cooling, the dynamically disordered high-temperature polymorphic Form I is transformed to the completely ordered low-temperature polymorphic Form III via the intermediate (transient) modulated phase II. This behavior is associated with a significant reduction in the kinetic energy of the rotating and flipping ester substituents, as well as a decrease in structural ordering into two distinct positions. In transient phase II, the conventional three-dimensional structure is modulated by periodic distortions caused by cooperative conformation exchange of the ester substituent between the two states, which is enabled by weakened hydrogen bonding. Based on solid-state NMR data analysis, the mechanism of the enantiotropic phase transition and the presence of the transient modulated phase are documented.

## 1. Introduction

Crystal polymorphism, a phenomenon that occurs in both organic and inorganic solids, is defined as the ability of a compound to crystallize in different crystalline phases. In general, this phenomenon is associated with different arrangements and/or conformations of molecules in the crystal lattice and is controlled by intermolecular interactions in the crystal lattice [1]. Since polymorphs differ in essentially every property, determining the correct form of a compound is very important in many areas of materials research. Especially in the pharmaceutical industry, special attention must be paid to the production of the desired crystal modification. This requirement stems from the fact that any undesirable polymorphic transformation can alter the solubility, bioavailability and/or chemical stability of the drugs produced. This is why polymorphism, a difficult-to-predict phenomenon, continues to be the focus of intensive research [2,3].

In this respect, dynamic processes caused by weakened intermolecular interactions in the crystal lattice must be seriously considered. This is because completely rigid molecules are rather rare and a significant fraction of organic compounds contain rotatable bonds and molecular units that allow conformational flexibility. In many cases, internal molecular motions are not completely quenched, even at low temperatures. Rather, it is reasonable to assume that dynamic processes have a substantial impact not only on the chemical reactivity, solubility and bioavailability of different polymorphs but also on the mechanism of polymorphic transitions.

**Figure 1:**
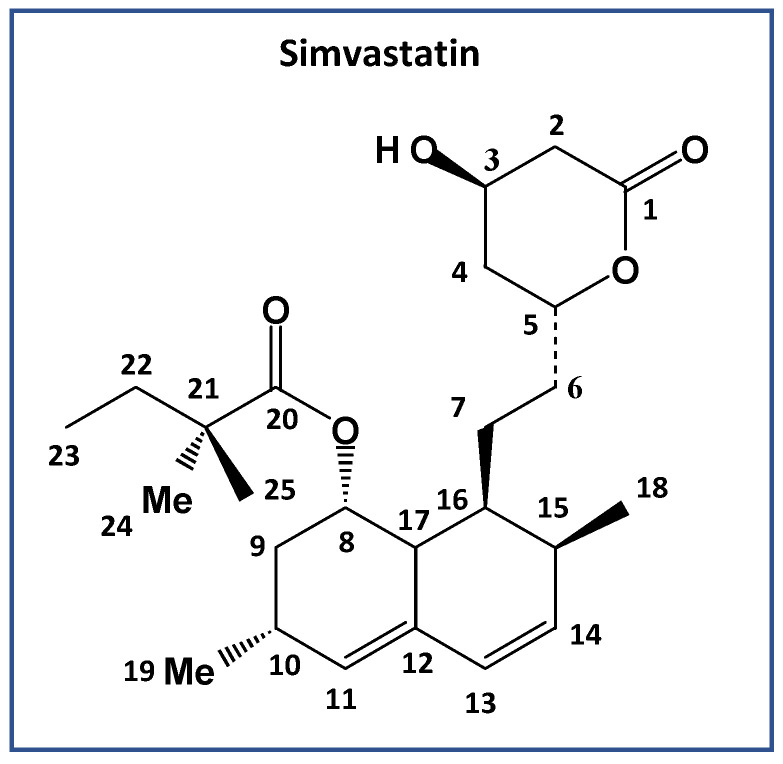
Chemical structure and numbering of simvastatin, C_25_H_38_O_5_.

A typical representative of such compounds is simvastatin (Figure 1). Although active pharmaceutical ingredients (APIs) have long been used to control hypercholesterolemia, new low-temperature polymorphic APIs were serendipitously discovered in 2010 [4]. Later in 2018, Simões et al. [5] investigated the three-fold polymorphism of simvastatin, exploiting X-ray, calorimetry and molecular-simulation methods to investigate the related structural, conformational and thermodynamic aspects. In the present study, we report a detailed analysis of simvastatin polymorphisms and focus on unravelling the dynamical nature of the disorder in its high-temperature polymorph. Particular attention is given to the interpretation of the segmental dynamics of its molecules in the crystal lattice, especially in the context of its two reversible structural transitions. In this respect, segmental dynamics are experimentally analyzed over a wide range of temperatures, thus providing information on motional frequencies and amplitudes. In light of the obtained information on segmental dynamics, the intermolecular interactions of all three polymorphs of simvastatin are evaluated with nuclear magnetic resonance (NMR) crystallography, which has already demonstrated its remarkable potential [6,7,8,9,10,11,12,13,14]. We believe that the presented data on the role of segmental dynamics in the formation of new polymorphic forms of simvastatin can provide general information for understanding phase transformations in solid systems with substantial entropy changes.

## 2. Results

### 2.1. Thermal Analysis

**Figure 2:**
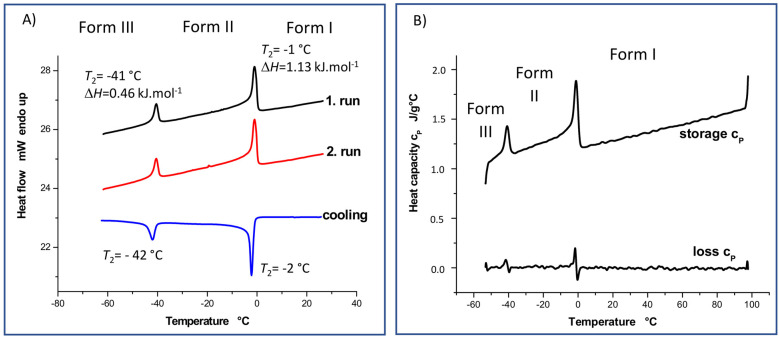
DSC (**A**) and DDSC (**B**) thermograms measured for simvastatin at temperatures ranging from −60 to +30 °C and from −60 to +100 °C, respectively.

Differential scanning calorimetry (DSC), which can be used to examine melting, crystallization and glass transition temperatures, heat capacity and enthalpy, is one of the most suitable thermal techniques for characterizing organic solids. The ease with which this technique can monitor the interconversion between different crystal modifications induced by energy added or removed during DSC experiments contributes to its high potential. Because metastable forms can spontaneously convert to more stable forms and vice versa, DSC measurements can reveal unknown crystal modifications. A DSC thermogram of simvastatin (Figure 2) shows two structural transitions occurring at *T*_1_ = 272 and *T*_2_ = 232 K (exothermic when cooled). These events are reversible, independent of the heating/cooling rate and exhibit negligible hysteresis. The observed processes correspond to solid–solid inter-conversions between three crystal modifications. As both these transitions are reversible, simvastatin exhibits enantiotropic behavior in the temperature range from 213 to 373 K.

The lack of temperature hysteresis and the specific λ shape are signatures of second-order phase transitions, which usually accompany the order–disorder type of transitions [15], although a λ-transition has also been observed recently for a disorder–disorder transition in molecular crystals [16]. It is interesting to see that the measured Δ*H* values associated with both these transitions are rather low (Δ*H* = 1.13 and 0.46 kJ.mol^−1^, at *T*_1_ and *T*_2_, respectively), which is consistent with the exceptionally small changes of the unit–cell parameters upon both these phase transitions. The observed phase-transition data can be further exploited to evaluate the entropy changes associated with both solid–solid phase transitions (Δ*S* = 4.15 and 1.98 J·mol^−1^·K^−1^, at *T*_1_ and *T*_2_, respectively). One can deduce from these entropy changes that the extent of disorder is increased more significantly upon the phase transition from Form II to Form I. Still, the respective disorder needs to be limited only to certain molecular segments as long as the melting of Form I into liquid is accompanied by a significantly larger entropy change of 77.96 J·mol^−1^·K^−1^ [17].

It is worth emphasizing that a significant decrease in the heat flow and heat capacity was observed above the transition temperature *T*_1_ (specific heat capacity, c_p_, is reduced by about 0.102 J·g^−1^·K^−1^, Figure 2), which is consistent with the findings of Simões et al. [16]. Such a decrease in heat capacity is usually explained by the increase in molecular order, typically occurring upon the crystallization of liquids or glasses. In this case, however, it would be surprising if a higher-order system (simvastatin, Form I) occurred at higher temperatures, while the low-temperature forms of simvastatin would be considered to be more disordered. Based on the calorimetric heat capacities for individual polymorphs, the entropy changes due to heating of the Form II within the whole temperature interval of its thermodynamic stability, thus from *T*_2_ to *T*_1_, can be evaluated to 81.5 J·mol^−1^·K^−1^. For comparison, an analogous entropy change in the same temperature interval can be estimated to only 71.6 J·mol^−1^·K^−1^. A possible explanation of this unusual behavior of heat capacities and entropies might indeed arise from the disordered nature of the Forms II and I. Taking the close structural similarity of all three simvastatin polymorphs into account, this appreciably larger entropy increase in the heated Form II could be explained through the gradual temperature-induced turning on of the dynamic disorder on Form II, which is discussed below.

Still, the high-temperature Form I needs to exhibit the highest entropy among the polymorphs above *T*_1_, which can appear to contradict the observed heat capacity trends. Significant crossovers of the heat capacities of the polymorphs at lower temperatures and/or a more important residual entropy inherent to the Form I can explain why its entropy is the highest, although it is barely possible to verify these hypotheses experimentally due to the minimal hysteresis of the polymorphic phase transitions.

### 2.2. Variable-Temperature ^13^C CP/MAS NMR Spectroscopy

**Figure 3:**
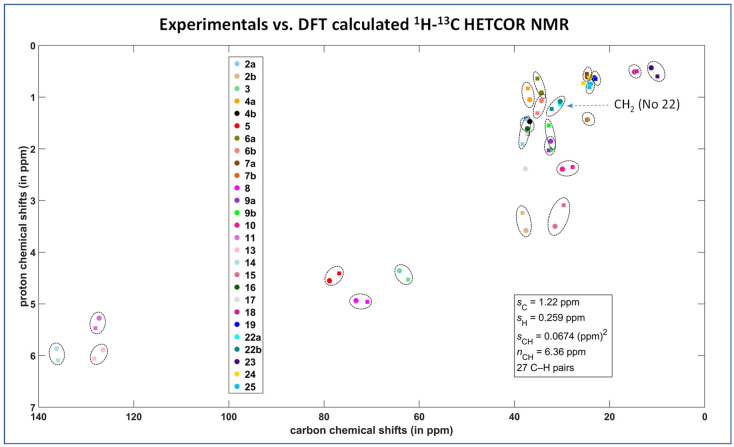
Comparison between theory and experiment for the ^1^H–^13^C HETCOR spectrum of Form I of simvastatin. The measured and predicted data are marked with squares and circles, respectively (the atom numbering is detailed in Supplementary Materials, Tables S1 and S2). Statistical parameters, which are shown in the inset, are specified in the Section 5.

In our previous work [18], using the combination of spectral editing and two-dimensional correlation techniques including ^13^C cross-polarization polarization-inversion (CPPI), solid-state attach-proton-test (SoS APT) and the transfer-dephasing-optimized refocused ^13^C CP-INADEQUTE experiment, we achieved a complete signal assignment of ^13^C NMR resonances of Simvastatin Form I. Subsequently, using the ^1^H–^13^C through-bond and through-space HETCOR experiments we identified the resonance frequencies of the corresponding hydrogen atoms. In our current work, in order to verify the proposed signal assignment, the previous experimentation was supplemented by GIPAW DFT calculations of the ^13^C and ^1^H shielding parameters which are summarized in the Supplementary Materials (see Tables S1 and S2, and Figures S1 and S2; and the resulting MAGRES files and PDB files). In this regard, the overlay of the calculated and experimental ^1^H–^13^C correlations shows that all ^1^H and ^13^C resonances are appropriately mapped out and the proposed signal assignment is reliable and correct (Figure 3).

**Figure 4:**
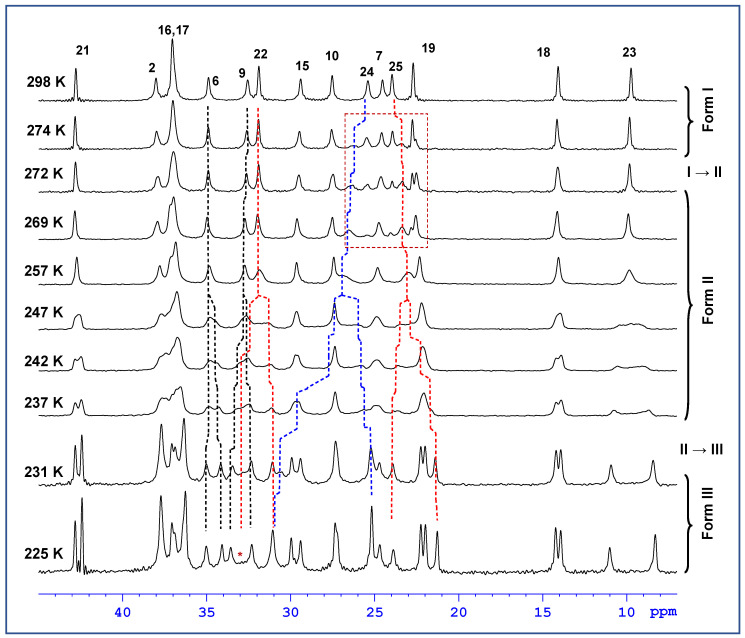
Expanded part of the variable temperature ^13^C CP/MAS NMR spectra of simvastatin, measured in the temperature range 231–298 K. Asterix * indicates position of a broadened signal.

**Figure 5:**
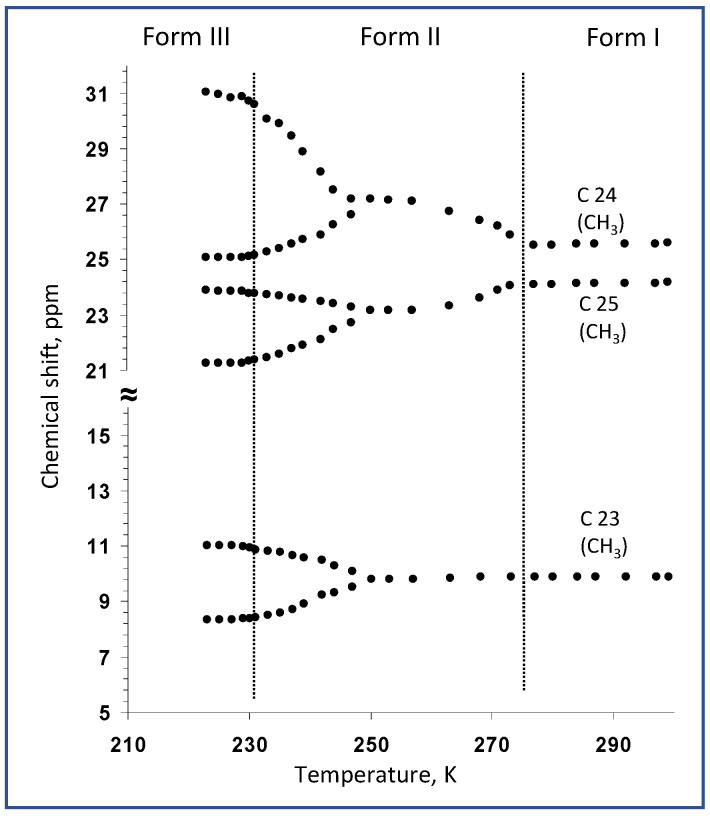
Temperature dependences of ^13^C NMR chemical shifts of carbons of the ester tail, measured in the temperature range of 300 to 220 K.

In the next step of our investigation, on the basis of the complete assignment of ^13^C NMR resonances, the variable-temperature ^13^C CP/MAS NMR spectra clearly identified the molecular sites whose local environment was considerably affected by phase transformations. At room temperature, the recorded ^13^C CP/MAS NMR signals of simvastatin were narrowed, with a linewidth mostly below 20 Hz. This indicates a highly ordered system without static structural defects (Form I). During cooling, a new set of ^13^C NMR signals appeared at approximately 274–269 K (Figure 4), indicating the formation of a new Form II crystal. In the temperature range of 272–232 K, the ^13^C resonance frequencies exhibited a strong temperature dependence, while the signals of methyls in the ester substituent (C23–C25) showed extensive broadening. Below approximately 247–242 K, all the detected NMR signals split into broad doublets whose resonance frequencies exhibited a pronounced temperature dependence (Figure 5). The signal of carbonyl carbon C1 was the only exception, suggesting that the local structure in this part of the simvastatin molecule was thermally stable. The signal broadening and temperature-induced splitting then clearly revealed an extensive conformation exchange process. As the temperature continued to decrease, the process of signal broadening was reversed, and the signals exhibited a distinct narrowing around the second transition temperature (*T*_2_ = 232 K). Below this temperature, the ^13^C NMR signals no longer exhibited considerable temperature dependences, and the two detected sets of ^13^C CP/MAS NMR signals corresponded to two symmetry-independent molecules in the low-temperature Form III.

The crystal structures of simvastatin were optimized by plane-wave density-functional theory (PW DFT) calculations, and their NMR chemical shielding was predicted using the gauge-including projector augmented wave (GIPAW) approach, as detailed in the Materials and Methods section. Importantly, the isotropic ^13^C and ^1^H chemical shieldings obtained by the GIPAW method for the PW DFT structure of the Form I crystal agreed well with the corresponding experimental ^13^C and ^1^H chemical shifts: the linear regression model had a standard deviation of 1.45 ppm and an adjusted *R*^2^ value of 0.99927 for ^13^C and 0.25 ppm and 0.97869 for ^1^H (the data are listed in the Supplementary Materials Tables S1 and S2 and Figures S1 and S2, respectively). The PW DFT geometries were used to obtain the structural parameter values discussed below.

Although the interconversion between crystal forms is a first-order transition that should occur instantly within a narrow temperature region according to thermodynamics, the recorded ssNMR spectra revealed much more gradual and complex processes. At first, the apparent coexistence of two crystal modifications (Forms I and II) in the temperature region 274–268 K around the transition I → II (Figure 4, red box) might be explained by a temperature gradient within the sample. Temperature gradients within the sample induced by the frictional heating of rapidly rotating rotors have been shown to reach up to 12 K at a spinning speed of 17 kHz [19]. At the spinning speed of 10 kHz applied during variable-temperature experiments, the gradient reached approximately 4–5 K, which corresponds well with the temperature region of the apparent coexistence of both crystal modifications. In this regard it is worth mentioning that the temperature gradient normally reaching up to ca. 12 K at 17 kHz in a 4 mm rotor can be reduced to ca. 1 K by applying the KelF spacer [19]. On the other hand, by using this spacer the amount of the sample is also significantly reduced, which would lead to a substantial increase in the experimental time required to reach an acceptable signal-to-noise ratio. Therefore, we decided to only reduce the spinning frequency to keep the temperature gradient in the acceptable limits mentioned above.

On the other hand, as indicated by the strong temperature dependences and severe broadening of some ^13^C NMR signals, extensive dynamic processes occurred not only in crystalline Form II but also in Form III. This was due to the asymmetry of the observed doublets. The asymmetric behavior of the individual components of the doublets was particularly apparent for the signals of the C22 methylene units in the nonequivalent molecules of simvastatin. While the component of the doublet resonating at approximately 31 ppm was relatively narrow, the second component at approximately 37 ppm was near the detection limit (Figure 4, see the asterisk in the spectrum measured at 225 K). This unusual broadening has been detected and described for crystalline poly(ethylene oxide) when the competition between segmental motions and dipolar decoupling leads to the destructive interference of NMR signals [20,21,22]. Thus, this finding indicates the existence of some residual motional disorder, even at temperatures below 220 K. Consequently, to better understand all the observed changes in ^13^C NMR chemical shifts, segmental dynamics, including motional frequencies and amplitudes, must be thoroughly investigated.

### 2.3. High-Frequency Motions

It has long been known that, in addition to their structure, molecular dynamics determines the physical properties of organic substances over a wide time range, from picoseconds to seconds. High-amplitude motions have been found in both amorphous systems and seemingly rigid crystallites [6,23]. Thus, the analysis of segmental dynamics is an important step toward understanding any solid–solid phase transitions. In this regard, two distinctive types of local motions are easily distinguishable via nuclear spin relaxation measurements: (i) high-frequency motions (librations, rotations and jumps of small groups with frequencies of approximately hundreds of MHz), which are probed by measurements of *T*_1_ relaxation times in the laboratory reference frame; and (ii) medium-frequency motions (approximately tens of kHz) involving movements of larger molecular segments, which are monitored by *T*_1ρ_ relaxation experiments in the rotating frame. The temperature dependences of the determined relaxation times provide the most information. Ideally, a logarithmic plot of relaxation rates (ln(1/*T*_1_)) vs. inverse temperature (1/*T*) should exhibit an ∧-shaped curve with a maximum at the temperature where the relaxation mechanisms are the most effective. This is roughly reached when the segmental motion frequencies are close to the resonance frequency of the relaxing nucleus (e.g., 125 MHz for ^13^C at 11.7 T, *T*_1_ relaxation times) or to the frequency of the rotating *rf* field applied for spin locking (usually 10–100 kHz, *T*_1ρ_ relaxation time). Furthermore, using the simplified approach, it is assumed that if the relaxation time increases with increasing temperature, the segmental motions are fast (correlation frequencies are higher than the resonance frequency), and that if the relaxation time decreases with increasing temperature, the internal motions are relatively slow. According to a simple Arrhenius function 1/*T*_1_ = A·exp(E*/*RT*) the activation energies (E*) of the segmental motions are then obtained from the slope of the linear parts of these dependences.

**Figure 6:**
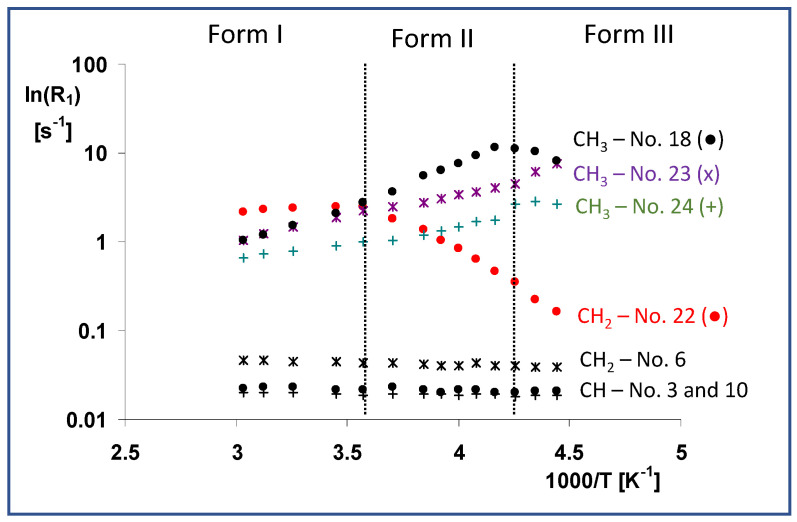
Temperature dependences of *T*_1_(^13^C) relaxation times of the selected units (CH—No. 3 and 10, CH_2_—No. 6 and 22 and CH_3_—No. 21, 22, and 25), measured in the temperature range of 330 to 220 K. These dependences are expressed as ln(*R*_1_) vs. inverse temperature 1/*T*, where the relaxation rate is defined as *R*_1_ = 1/*T*_1_(^13^C).

According to the abovementioned rules, the variable-temperature *T*_1_(^13^C) relaxation measurements reveal that the lactone ring and bicyclic part of the simvastatin molecule were rigid in the high-temperature crystal Form I. Additionally, the CH_2_ groups in the ethylene linker (C6, see Figure 6) were unlikely to have high segmental dynamics.

On the other hand, very short *T*_1_ relaxation times ranging from 0.4 to 1.1 s can directly identify molecular segments undergoing high-frequency motions. As expected, these are predominantly methyl groups and fast rotation and three-site jumps have been observed even in crystalline solids. For instance, assuming three-site jumps were the dominant motion and applying a procedure recently used by J.M. Njus [24], we determined the angular correlation times *τ*_c_ of the methyl groups, which ranged from 4.8 × 10^−11^ to 1.1 × 10^−10^ s under standard laboratory conditions. In addition to the methyl groups, however, the whole ester tail moves quickly with a comparable correlation frequency, as indicated by the very short relaxation times (0.4–0.5 s) of the corresponding methylene group (C22). According to a rough estimate based on the linear but still rising temperature dependence of the relaxation rate, the dominant motion of the whole ester tail in crystal Form I had a correlation time of less than 8.0 × 10^−9^ s. The first crystal phase transition (Form I → Form II), however, did not dramatically change the motional behavior of the methyl groups, and their correlation times increased monotonically, reaching 9.5 × 10^−11^–6.3 × 10^−10^ s at 240 K. Similarly, the corresponding activation energies were nearly unchanged (Figure 6).

In contrast, and according to the Arrhenius function mentioned above, the very fast and almost unrestricted motion of the whole ester tail in Form I was characterized by the low activation energy of the motion of the ester CH_2_ group (carbon atom C22), E* = 4.1 kJ·mol^−1^, which became dramatically restricted after the transition to Form II, when the activation energy reached E* = 30.0 kJ·mol^−1^. The reversal of the temperature dependence of the relaxation rate indicates that the correlation time of the ester tail motion was greater than approximately 8.0 × 10^−9^ s (it increased with decreasing temperature).

The unchanged trend in the sharp decrease in *T*_1_ relaxation times of the ester CH_2_ group (carbon atom C22) was also apparent after the second phase transition from crystal Form II to Form III. Although the relatively long *T*_1_ (^13^C) relaxation time at 225 K (6.1 s) indicates gradually decreasing dynamics, the high-frequency motions of the ester tails persisted even below this relatively low temperature. In addition, the transition to crystal Form III was accompanied by considerable changes in the dynamics of the methyl rotations. The observed inversion of the temperature dependence of the relaxation rates (methyl carbons C18 and C24) indicates that the correlation times of their rotation had increased to greater than 8.0 × 10^−9^ s.

### 2.4. Medium-Frequency Motions

**Figure 7:**
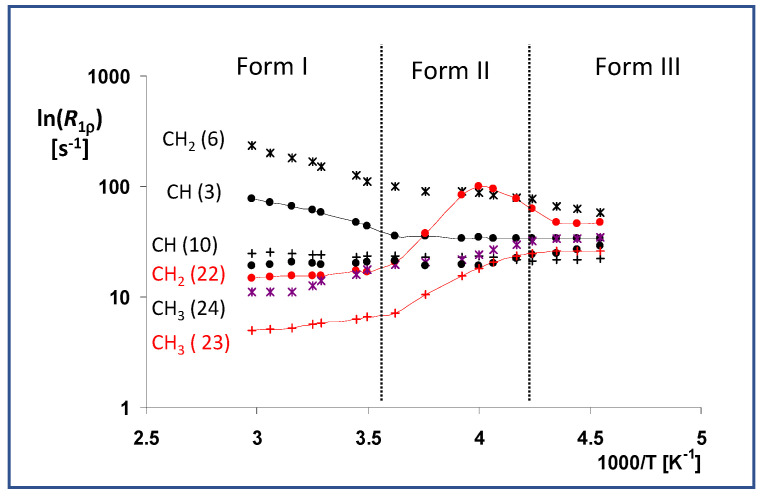
Temperature dependences of the *T*_1ρ_ (^13^C) relaxation times of the selected units (CH—No. 3 and 10, CH_2_—No. 6 and 20 and CH_3_—No. 21, 22, and 25), measured in the temperature range of 330 to 220 K. These dependences are expressed as ln(*R*_1ρ_) vs. inverse temperature 1/*T*, where the relaxation rate is defined as *R*_1ρ_ = 1/*T*_1ρ_(^13^C).

When we analyzed the relatively slow segmental motions, we found that the methylene group atom C22 and the neighboring methyl C23, both localized in the ester tail, exhibited remarkable motion activity. These units, specifically those in metastable transient modification II, executed strongly thermally activated segmental motions. Moreover, the obtained temperature dependence and maximum relaxation rate *R*_1ρ_ (Figure 7) indicate that the overall correlation time of this motion at 250 K was close to approximately 16 μs (1/62.5 kHz, intensity of spin locking field). It is worth noting that the observed maximum relaxation rate corresponded to the splitting/coalescence temperature of the ^13^C NMR signals. In the low-temperature Form III, these motions appeared to be considerably quenched deep in the energy depression, exhibiting negligible thermal activity. On the other hand, the weak thermal dependence of the relaxation rate *R*_1ρ_ observed for these units (C22 and C23) in high-temperature Form I indicates that all the energy barriers had already been overcome, thus allowing free unhindered rotation motions for the whole ester group. In this regard it is worth noting that the obtained experimental ss-NMR relaxation data are in good agreement with the previously published molecular dynamics simulations [5,25].

### 2.5. Motional Amplitudes

**Figure 8:**
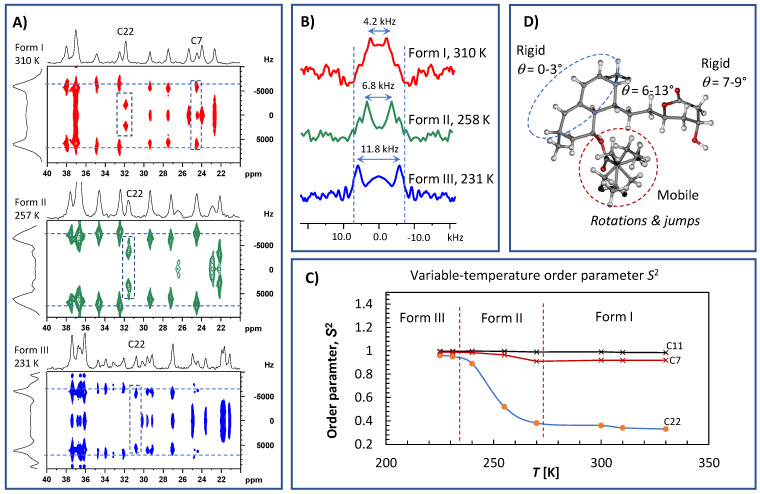
Expanded parts of ^1^H–^13^C PILGRIM NMR spectra of crystalline simvastatin measured at 310, 257 and 231 K (**A**); the corresponding ^1^H–^13^C dipolar profiles extracted for methylene CH_2_ unit C22 (**B**); the plot of selected order parameters *S*^2^ vs. temperature (**C**); and the molecular structure of simvastatin displaying different segmental motions (**D**). The corresponding full range ^1^H–^13^C PILGRIM NMR spectra are presented in Supplementary Materials Figures S5–S7.

A description of molecular dynamics would not be complete without knowledge of the motional amplitudes of individual segments. Recent developments in recoupling techniques have provided new ways to probe the geometry of segmental motion. In particular, ^1^H–^13^C separated local-field experiments [26,27,28,29] have enabled straightforward site-specific measurements of dipolar couplings that directly contain this information. This is because any molecular motion with a correlation time of less than approximately 40 μs causes the averaging of one-bond ^13^C–^1^H dipolar interactions. Consequently, the ^1^H–^13^C spin–pair dipolar interactions in the CH and CH_2_ units in powdered solids produce Pake’s doublets, with peak-to-peak splitting reflecting the effective dipolar coupling constants *D*_CH_ [26,27,28,29,30,31]. Assuming that the C–H chemical bonds have a constant length, the reduced splitting of the ^1^H–^13^C dipolar spectra relative to the theoretical rigid limit value, *D*_CH,rig_, indicates the presence of segmental motion. The rigid limit value of one-bond ^13^C–^1^H dipolar interactions is ca. 23.5 kHz. However, under the applied experimental conditions (see Materials and Methods), the rigid-limit value was scaled down by a scaling factor of cos(54.7°) to be ca. 13.2 kHz. The one-bond dipolar spectra could easily be extracted as vertical slices from the 2D ^1^H–^13^C PILGRIM spectra shown in Figure 8A. The analysis of the site-specific dipolar profiles measured at temperatures ranging from 220 to 340 K (Figure 8B) enabled the motional amplitudes of each segment to be mapped over a wide temperature range and for different crystal modifications.

In this way, four parts of the simvastatin molecule with distinct motional amplitudes were revealed (Figure 8C). Specifically, all CH and CH_2_ segments of the bicyclic fragment were found to exhibit motionally averaged dipolar couplings, *D_CH_*, of approximately 13.3–13.5 kHz, which are very close to the values expected for completely rigid segments (*D*_CH,rig_ = 13.2–13.5 kHz; the experimental uncertainty ±0.1 kHz corresponds to the FID resolution in indirect *t*_1_ dimension). Consequently, the related order parameter, $S_{CH}^{2}$, which is defined as the ratio of the motionally averaged dipolar coupling constant to the rigid-limit value, ranged between 0.98 and 1.00. In the simplest case, if the segmental motion is axially symmetric and small in amplitude (for a fluctuation angle *θ*, ⟨sinθ⟩=⟨θ⟩), the order parameter can be converted to a root-mean-square angular fluctuation angle, $\sqrt{\left\langle \theta^{2}\right\rangle }$, according to the following definition: $S_{CH}^{2}=1-\frac{3}{2}\langle \theta^{2}\rangle$. In contrast, for the high-amplitude discrete jumps between *N* distinct orientations, the order parameter is defined as $S_{CH}^{2}=\sum_{i,j=1}^{N}p_{i}p_{j}P_{2}\left(cos\theta_{ij}\right)$ in which *p* is the equilibrium population of vector having the *i*-th orientation and *θ_ij_* is the angle between vectors having the *i*-th and *j*-th orientations (see [31,32] for more details). This indicates that the bicyclic fragment of the simvastatin molecule is rigid, with nearly negligible reorientation amplitudes and an average fluctuation angle close to zero degrees (between approximately 0 and 5°). It is worth noting that this behavior is temperature independent and occurs in all three crystal modifications of simvastatin. The CH and CH_2_ groups of the lactone cycle showed slightly higher flexibility, as measured by motional amplitudes ranging from 7 to 9° ($S_{CH}^{2}$ = 0.97–0.96). Additionally, in this case, the observed low-amplitude wobbling of the lactone segment was essentially temperature independent and remained constant for all three crystal modifications.

A slightly surprising temperature dependence of motion amplitudes was found for the ethylene linker (C7) connecting the lactone ring with the bicyclic unit. During the first temperature transition, the low-amplitude fluctuations of approximately 12–13° at 310 K decreased to approximately 6–8° at 260 K (Figure 8D), whereas below 235 K in crystal Form III, these motions almost completely disappeared ($S_{CH}^{2}$ = 0.98–0.99).

As indicated by the spin–relaxation behavior, the most flexible part of simvastatin is its ester tail. This was clearly reflected by the very low-order parameter of the ethyl CH_2_ group C22, which was approximately $S_{CH}^{2}$ = 0.33–0.37 and remained nearly constant over the temperature range of 320 to 275 K. This low value of the order parameter determined for the high-temperature crystal modification I shows that the ester substituent undergoes unrestricted, fast, large-amplitude rotations and jumps [32]. We assume that the rotation motions around the C20-C21 and C22-C21 axes occurred simultaneously. It is interesting that this motion had a negligible impact on the DFT GIPAW calculations of ^1^H and ^13^C NMR isotropic chemical shifts as demonstrated in Figure 3 (the region of the correlation of CH_2_ group No. 22 is highlighted by an arrow).

In contrast, below the first transition temperature (273 K), i.e., in crystal modification II, these motions exhibited a very strong temperature dependence, which was reflected in the steep increase in the order parameter from approximately 0.38 at 270 K to approximately 0.89 at 240 K. This indicates that nearly free rotation motions were gradually hindered, resembling discrete two-site jumps. This idea is further supported by signal splitting, which was clearly visible below approximately 255 K. Finally, below the second transition temperature (231 K), the order parameter was less temperature dependent, with values ranging from approximately $S_{CH}^{2}$ = 0.91 to 0.97. This indicates that the ester group of at least one symmetry-independent molecule was immobilized and executed relatively low-amplitude motions (wobbling) with an average angular fluctuation angle of approximately 8–15°. Unfortunately, due to severe signal broadening, such a parameter could not be precisely determined for the ester unit of the second symmetry-independent molecule.

## 3. Discussion

Our NMR experimental results clearly reveal three distinct motional regimes in crystalline simvastatin that are separated by two fully-reversible solid–solid phase transitions in which ester substituents play a central role. At temperatures below the low-temperature transition (232 K), the simvastatin molecules are essentially rigid, with considerably restricted segmental dynamics that are practically temperature-independent. In contrast, above the high-temperature transition (272 K), the ester substituents exhibit extensive, unrestricted and highly symmetric uniaxial rotation motions. In this case, thermal activation is also very weak. The other parts of the simvastatin molecule remain basically rigid, undergoing only low-amplitude fluctuations. However, an intermediate phase II exists between the two temperature transitions, ranging from approximately 232 to 272 K, and shows strongly thermally activated segmental motions as well as dramatic structural rearrangement.

**Figure 9:**
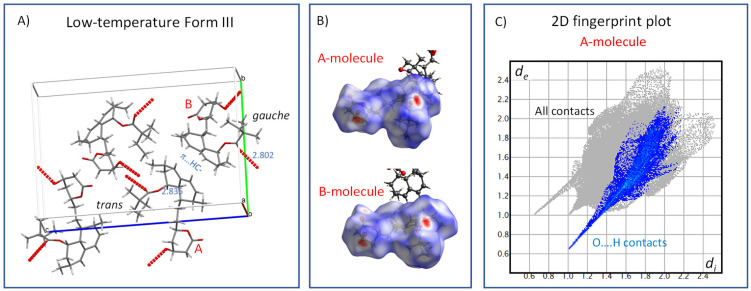
The crystal structure of the low-temperature Form III (150 K, CSD Entry: EJEQAL01) (**A**); Hirshfeld surface mapped over *d*_norm_, using a color scale of red (shorter than vdW separation), white (equal to vdW separation), and blue (longer than vdW separation) for the symmetry independent A and B molecules (**B**); and the corresponding 2D fingerprint plot (**C**).

To better understand the molecular mechanisms of the phenomena that separate individual crystal phases of simvastatin and control phase transitions, we first analyzed the crystal structure of the low-temperature Form III (Figure 9). Form III is distinguished by the presence of two symmetry-independent molecules (designated A and B below; see also reference [4]), which have different torsion angles in the ester parts C20-C21-C22-C23 and O4-C20-C21-C22 (A: −172° and −11°; B: −76° and −36°). Molecular packing is driven predominantly by hydrogen bonding involving ester-carboxyl groups C20 and hydroxyl groups of the lacton cycle [33]. Additional interactions that appear to play a role are C–H…π noncovalent bonding. It is worth noting that the strength of hydrogen bonding differs for both symmetry-independent molecules, as reflected by O…O distances of 2.835 and 2.802 Å for A and B molecules, respectively. The differences in H-bond interactions between the inequivalent molecules are clearly seen in the Hirshfeld surfaces and the corresponding fingerprints (Figure 9B,C, respectively). The −OH···OC and CO···HO− intermolecular contacts in A and B molecules typically appear as two distinct spikes close to the diagonal in their 2D fingerprint plots (Figure 9C; O…H interactions are selectively highlighted in right-hand plots, and the spikes show a vdW radius of approximately 0.6 Å, confirming tight contacts). In the Hirshfeld surface plots, these interactions are indicated by bright red spots [34].

**Figure 10:**
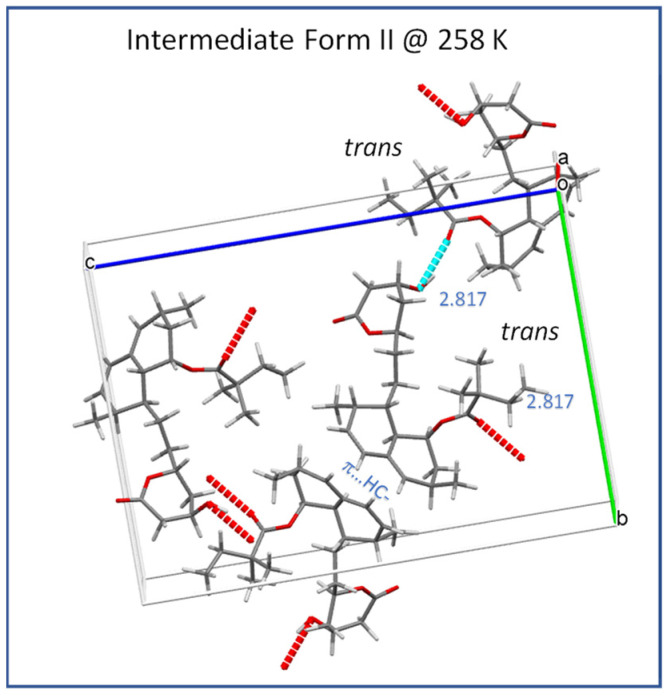
Crystal structure of the intermediate Form II (258 K; CSD Entry: EJEQAL02).

As indicated by the strong temperature dependence of the *T*_1_ (^13^C) and *T*_1ρ_ (^13^C) relaxation times of ester methylene carbon C22 (Figure 6 and Figure 7) observed upon heating above the low-temperature transition (232 K), the hydrogen bonding of ester substituents is considerably weakened and/or periodically interrupted in the intermediate Form II, thus allowing conformation exchange between molecules A and B. This exchange is evidenced by the gradual broadening and decreased separation of some pairs of ^13^C CP/MAS NMR signals after heating to approximately 250 K. The signal coalescence observed above this temperature indicates that the conformation exchange between molecules A and B becomes too fast to detect in the ^13^C CP/MAS NMR spectra. As this coalescence is not accompanied by any energetic event, according to the DSC results, no phase transition can be attributed to the observed splitting coalescence of the signal. Thus, the intermediate phase II, depending on the temperature and time window of the observation, may involve one or two symmetry-independent molecules. The low-temperature transition event is thus associated with overcoming the energy barriers required for temporal breaking or the weakening of hydrogen bonding and trans-gauche conformation transitions. Figure 10 shows a typical time-averaged crystal structure of Form II at 258 K, where the hydrogen bonding between the ester carboxyl C=O and lactone hydroxyl HO− group is uniform and described by an O…O distance of 2.817 Å.

**Figure 11:**
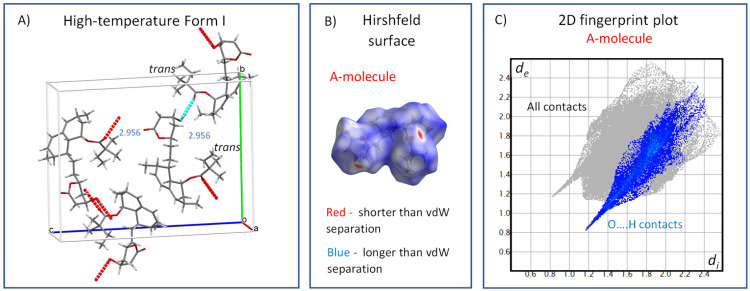
The crystal structure of the high-temperature Form I (300 K, CSD Entry: EJEQAL); (**A**); Hirshfeld surface mapped over *d*_norm_, using a color scale of red (shorter than vdW separation), white (equal to vdW separation) and blue (longer than vdW separation) for the single molecule (**B**); and the corresponding 2D fingerprint plot (**C**).

Upon further heating, i.e., above the high-temperature transition (272 K), the unit cell volume increased from 2358 to 2382 Å^3^, which indicates a decreased density, the additional weakening and reconstitution of hydrogen bonds and an increase in the free crystal volume. The average length of the hydrogen bonds in the high-temperature Form I was 2.956 Å (Figure 11A), and the weakening of hydrogen bonding can be clearly seen in the Hirshfeld surfaces and corresponding fingerprints (Figure 11B,C, respectively). The red dots were less intense, whereas the spikes corresponding to O…H contacts were shorter and shifted toward higher values of approximately 0.8 Å. The ester substituents occupying the *trans* conformation underwent extensive, unrestricted and highly symmetric uniaxial rotation motions along the C20–C21 axis. This indicates that above this transition, all energy barriers of conformation exchange and overall rotation were overcome. The resulting segmental motion was so fast and symmetric that the corresponding ^13^C CP/MAS NMR resonances were extremely narrow, resembling liquid isotropic motions. This type of motion-induced effective time-average ordering of the system can hence explain the slightly unusual decrease in heat flow and heat capacity observed above the first transition temperature of 272 K (Figure 2).

According to thermodynamics, the significant increase observed in segmental dynamics, which gradually occurred in the Form II when going from the Form III to Form I, consumes additional heat, causing the higher observed heat capacity of the Form II. This can also be regarded as an analogy to the heat capacity contribution of a hindered rotor, which typically rises steeply at low temperatures to exhibit a maximum and to converge from above to the high-temperature limit ½ R, valid for the heat capacity of a free rotor [35]. Provided that the ester moiety rotates nearly freely in the Form I, whereas a significant barrier to this motion is imposed in Form II, the observed lower heat capacity of the Form I arises from the fact that the segmental dynamics in Form I do not need to be further excited upon heating. Such a hierarchy of heat capacities of various polymorphs is rather unusual, but it typically occurs for rotationally disordered systems [36]. In these systems the fast time-scale dynamics impose a conformational entropic free energy penalty if the ester substituent is rigidified [31]. Consequently, the entropic contribution associated with the transition from the low-temperature polymorphic Form III to the high-temperature Form I must be considered as a key factor that stabilizes high-temperature crystal forms, especially given the weakened hydrogen bonds. As a result, polymorphs of simvastatin are not only associated with variations in molecular conformation or packing but also with changes in segmental dynamics, which must also be seriously considered.

**Figure 12:**
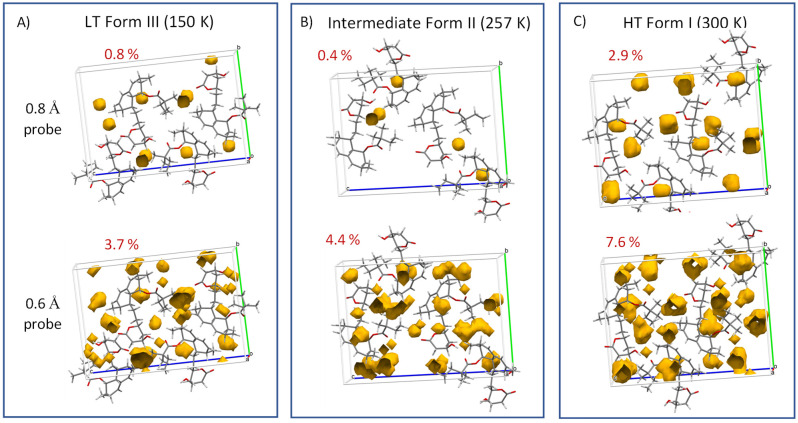
Free volume calculated for low-temperature Form III (**A**), intermediate Form II (**B**) and high-temperature Form I (**C**). Specifically, the empty spaces in the crystal unit cells large enough to hold spherical probes with radii of 0.8 or 0.6 Å are displayed.

Finally, when analyzing extensive high-amplitude segmental motions, the crystal-free volume must be considered. In this regard, the fraction of the free volume calculated for the intermediate Form II appeared to be particularly low. Specifically, empty spaces in the crystal unit cell large enough to hold spherical probes with radii of 0.8 or 0.6 Å occupied 0.4 or 4.4% of the free volume, respectively (see Figure 12). This finding thus suggests that high-amplitude jumps and conformation exchange require some form of cooperativity when several molecules simultaneously execute the same phase-coherent motion. The presence of such periodic changes, which occur when groups of atoms are shifted or rotated with respect to their neighbors, indicates the existence of a transient modulated intermediate phase [37].

## 4. Conclusions

In summary, on the basis of extensive solid-state NMR data analysis, the mechanism of the enantiotropic phase transition and the presence of the transient modulated phase was documented. It was clearly demonstrated that in crystalline molecular solids, in the absence of strong intermolecular interactions, entropy-driven processes play a key role in the formation of dynamically modulated transient phases. Specifically, in crystalline simvastatin, the observed fully reversible enantiotropic behavior is associated with multiple order–disorder transitions: upon cooling, the dynamically disordered high-temperature polymorphic Form I is transformed to the completely-ordered low-temperature polymorphic Form III via the intermediate (transient) modulated phase II. This behavior is associated with a significant reduction in the kinetic energy of the rotating and flipping ester substituents, as well as a decrease in structural ordering into two distinct positions. In the transient phase II, the conventional three-dimensional structure is modulated by periodic distortions caused by the cooperative conformation exchange of the ester substituent between the two states, which is enabled by weakened hydrogen bonding. In general, in the transient forms, the conventional three-dimensional periodic structure is modulated by periodic distortions, which cause changes in key physical properties. Therefore, the discovery of the transient modulated phase for crystalline simvastatin increases the need for the further investigation of molecular crystalline solids with bulky, potentially movable molecular segments.

## 5. Materials and Methods

Sample preparation. *n*-heptane (6.5 mL) was added to a solution of simvastatin (Biocon Limited, Bengaluru, India, in acetone (1.1 g per 3.5 mL). The mixture was allowed to stand overnight, and the resulting crystals were washed with *n*-heptane. A powdered sample (crystal Form I) was used for further experiments without any purification. The other forms were obtained by cooling the sample below 0 °C (Form II) and −41 °C (Form III).

Differential Scanning Calorimetry (DSC). DSC was carried out on a Perkin-Elmer PYRIS 1 system. Aluminum sample pans were used with approximately 10 mg of the sample. The sample was cooled (cooling rate 40 °C/min) from the laboratory temperature to −60 °C and tempered for 10 minutes. Then, the sample was heated (heating rate 10 °C/min) to 30 °C (1st run). Before the 2nd run, the sample was cooled to −60 °C (cooling rate 10 °C/min). Dynamic DSC (DDSC) was performed in the temperature range of −50 to 100 °C. A single point calibration was carried out using indium (meting point 156.60 °C) as a standard sample. Nitrogen was used as a purge gas in an ambient mode.

Solid-state NMR spectroscopy (ssNMR). SSNMR spectra were measured at 11.7 T using a Bruker Avance III HD 500 US/WB NMR spectrometer (Karlsruhe, Germany, 2013) in 4 mm ZrO_2_ rotors. The magic angle spinning (MAS) speed was 11 kHz, the nutation frequency of the *B*_1_ (^13^C) field for the standard cross-polarization experiment was 62.5 kHz, the contact time was 2 ms and the repetition delay was 4 s. Standard TPPM (two-pulse phase-modulated) dipolar decoupling was applied during the detection period. The phase modulation angle was 15° and the flip-pulse length was 4.2 μs. The applied nutation frequency of the *B*_1_(^1^H) field was *ω*_1_/2π = 89.3 kHz. High-quality spectra were obtained by accumulating 128 scans. To measure the *T*_1_ and *T*_1ρ_ relaxations of ^13^C magnetization, standard relaxation experiments [23] with cross polarization (CP) were performed. The intensity of the spin-locking field *B*_1_ (^13^C) expressed in frequency units *ω*_1_/2π = *γB*_1_ was 62.5 kHz, with durations ranging from 0.1 to 15 ms. Site-specific measurements of one-bond ^1^H–^13^C dipolar couplings under Lee–Goldburg conditions [38] were performed using PILGRIM experiments [26]. The length of the polarization–inversion period was 1 ms. Lee–Goldburg cross polarization was incremented from 50 to 2610 μs with 20 μs increments. The recycle delay was 2 s and the *t*_1_ evolution period consisted of 128 increments each made of 64–128 scans. Two-dimensional (2D) ^1^H-^13^C HETCOR experiments [39] were performed using the FSLG (Frequency Switched Lee–Goldburg) decoupling during the *t*_1_ evolution period consisting of 128 increments each made of 64–128 scans with a dwell time of 42.6 μs. Further experimental details can be found in our previous work [5]. All the variable temperature experiments were conducted between temperatures of 330 and 220 K. Frictional heating [19,40] of the spinning samples was compensated for by active cooling. The ^13^C CP/MAS NMR spectra were referenced to α-glycine (176.03 ppm—CO signal). The ^1^H scale was calibrated and scaled with the external standard—alanine low-field NH_3_ signal at 8.5 ppm and the high field CH_3_ signal at 1.2 ppm.

DFT Calculations. The PW DFT approach, which is detailed in [41,42,43], was used as implemented in the CASTEP 6.1 suite of codes [43]. The initial geometries of Forms I and III were taken from the Cambridge Crystallographic Data Centre (ref. codes EJEQAL and EJEQAL01, respectively). They were subjected to the optimization of all atomic positions, while the unit–cell parameters were kept at experimental values [4]. Ultrasoft on-the-fly generated pseudopotentials [44] and the Perdew–Burke–Erzherhof (PBE) [45] DFT functional were adopted. For the optimized crystal structures, the NMR chemical shielding tensors were predicted using the GIPAW strategy [46,47] in conjunction with the PBE functional (see reference [48] for a thorough comparison of the relevant computational techniques). In all CASTEP calculations, the settings were consistent with the “Fine” accuracy level of Materials Studio 5.0 software [49]. Specifically, the following Monkhorst–Pack grids [50] were employed in the calculations of Forms I and III, respectively: 4 × 1 × 1, 2 *k* points and 4 × 2 × 1, 2 *k* points. In all the calculations, the cut-off energy of the plane waves was set to 550 eV. Resulting MAGRES files and PDB files with atoms of asymmetric units are included in the Supplementary Materials. Similarity measures of the simulated 2D HETCOR spectrum were obtained by the previously developed procedure [51,52,53].

## Data Availability

The data presented in this study are available in the article and in the Supplementary Materials.

## Supplementary Materials

The following are available online: Table S1: Listing of the 13C chemical shift, δ and shielding, σ, data of Form I; Figure S1: The fit of δ and σ data from Table S1; Table S2: Listing of the 1H chemical shift, δ (experimental values), and shielding, σ (calculated values), data of Form I.; Figure S2. The fit of δ and σ data from Table S2; Figure S3: 1H–13C FSLG HETCOR NMR spectra of the high-temperature Form I of simvastatin measured at 300 K and 100, and 900 μs of CP mixing period; Figure S4: 1H-13C FSLG HETCOR NMR spectra of the low-temperature Form III of simvastatin measured at 225 K and CP mixing periods of 700 and 1600 μs; Figure S5: Full-range and expanded 1H–13C PILGRIM NMR spectra with the signal labeling of crystalline simvastatin measured at 310 K; Figure S6: Full-range and expanded 1H–13C PILGRIM NMR spectra with the signal labeling of crystalline simvastatin measured at 257 K; Figure S7: Full-range and expanded 1H–13C PILGRIM NMR spectra with the signal labeling of crystalline simvastatin measured at 231 K; Files ‘FormI.MAGRES’, ‘FormIII.MAGRES’, and files containing atoms of the asymmetric units: ‘FormI.PDB’ and ‘FormIII.PDB’.

## Sample Availability

Samples of the compounds are not available from authors.
