# Supplementary material for: Enantiotropy of Simvastatin as a Result of Weakened Interactions in the Crystal Lattice: Entropy-Driven Double Transitions and the Transient Modulated Phase as Seen by Solid-State NMR Spectroscopy
Source: Molecules. 2022 Jan 20;27(3):679. doi: 10.3390/molecules27030679 (PMC8838109; doi:10.3390/molecules27030679)

Supplementary Materials for

Enantiotropy of Simvastatin as a Result of Weakened Interactions in the Crystal Lattice: Entropy-Driven Double Transitions and the Transient Modulated Phase as Seen by Solid-State NMR Spectroscopy

Table S1. Listing of the ^{13}C chemical shift, δ, and shielding, σ, data of Form I.

atom number [a]	δ (in ppm)	σ (in ppm)
1	170.66	–6.0672
2	38.31	133.7313
3	62.35	104.9721
4	37.17	134.5756
5	76.83	89.0942
6	35.16	137.2332
7	24.78	147.6891
8	70.91	95.1280
9	32.80	139.3343
10	27.76	141.9998
11	128.05	36.7684
12	133.98	29.8038
13	128.26	37.6726
14	135.91	27.0738
15	29.65	140.3348
16	37.34	134.0537
17	37.34	133.5969
18	14.30	158.3840
19	22.95	149.2310
20	180.28	–17.9695
21	43.02	126.3609
22	32.16	141.4941
23	9.95	162.1834
24	25.52	148.2828
25	24.25	148.3931

Footnotes to Table S1

[a] corresponding to Figure 1 of the main text

[b] as used in the fit shown in Figure S1

Figure S1. The fit of δ and σ data from Table S1. The red line is $\sigma = -1.0658\times\delta + 173.42$ ppm.

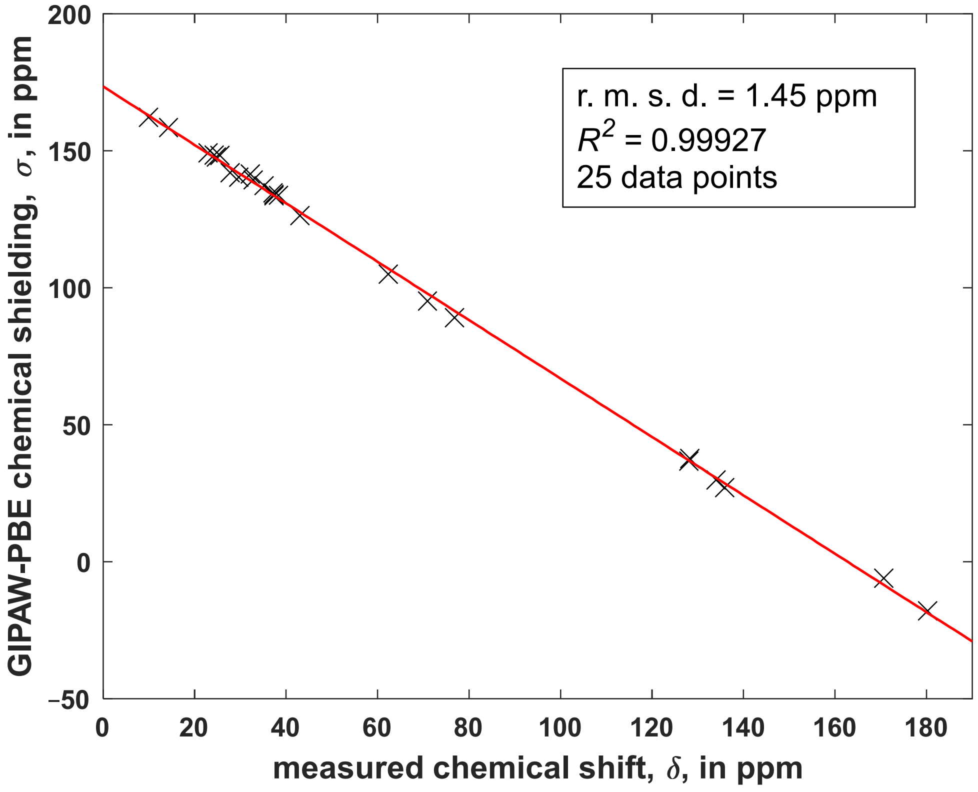

Table S2. Listing of the ^{1}H chemical shift, δ (experimental values), and shielding, σ (calculated values), data of Form I. The atom numbering is shown that corresponds to the 'FormI.MAGRES' and 'FormI.PDB' files in the Supplementary Materials.

proton #	designation	atom # in PDB file	atom # in MAGRES file	σ (in ppm) [a]	δ (in ppm) [a]
1	H2a	31	1	29.0661	1.91
2	H2b	32	2	26.9471	3.24
3	H3	33	3	26.1837	4.53
4	H4a	34	4	29.4299	1.71
5	H4b	35	5	29.0145	0.83
6	H5	36	6	25.9956	4.41
7	H6a	37	7	29.5610	0.64
8	H6b	38	8	29.4107	1.31
9	H7a	39	9	29.8624	1.44
10	H7b	40	10	29.0507	0.55
11	H8	41	11	25.6177	4.96
12	H9a	42	12	28.6420	2.03
13	H9b	43	13	28.4832	1.55
14	H10	44	14	28.1126	2.35
15	H11	45	15	25.2847	5.47
16	H13	46	16	24.6818	6.06
17	H14	47	17	24.7067	6.09
18	H15	48	18	27.0271	3.09
19	H16	49	19	28.8788	1.7
20	H17	50	20	28.1194	1.7
21	Met18	(51,52,53)	(21,22,23)	29.9560 [b]	0.50
22	Met18	(51,52,53)	(21,22,23)	29.9560 [b]	ditto
23	Met18	(51,52,53)	(21,22,23)	29.9560 [b]	ditto
24	Met19	(54,55,56)	(24,25,26)	29.8474 [c]	0.65
25	Met19	(54,55,56)	(24,25,26)	29.8474 [c]	ditto
26	Met19	(54,55,56)	(24,25,26)	29.8474 [c]	ditto
27	H22a	57	27	29.3485	1.23
28	H22b	58	28	29.3968	1.23
29	Met23	(59,60,61)	(29,30,31)	30.0351 [d]	0.60
30	Met23	(59,60,61)	(29,30,31)	30.0351 [d]	ditto
31	Met23	(59,60,61)	(29,30,31)	30.0351 [d]	ditto
32	Met24	(62,63,64)	(32,33,34)	29.8642 [e]	0.73
33	Met24	(62,63,64)	(32,33,34)	29.8642 [e]	ditto
34	Met24	(62,63,64)	(32,33,34)	29.8642 [e]	ditto
35	Met25	(65,66,67)	(35,36,37)	29.7258 [f]	0.81
36	Met25	(65,66,67)	(35,36,37)	29.7258 [f]	ditto
37	Met25	(65,66,67)	(35,36,37)	29.7258 [f]	ditto
38	hydroxyl	68	38	27.2823	3.2 (weak)

Footnotes to Table S2

[a] as used in the fit shown in Figure S2

[b] (30.1615+30.2996+29.4070)/3

[c] (29.6238+30.0353+29.8832)/3

[d] (29.7347+30.1323+30.2383)/3

[e] (30.0171+29.4951+30.0804)/3

[f] (29.5894+29.9253+29.6626)/3

Figure S2. The fit of δ and σ data from Table S2. The red line is $\sigma = -0.96127\times\delta + 30.413$ ppm.

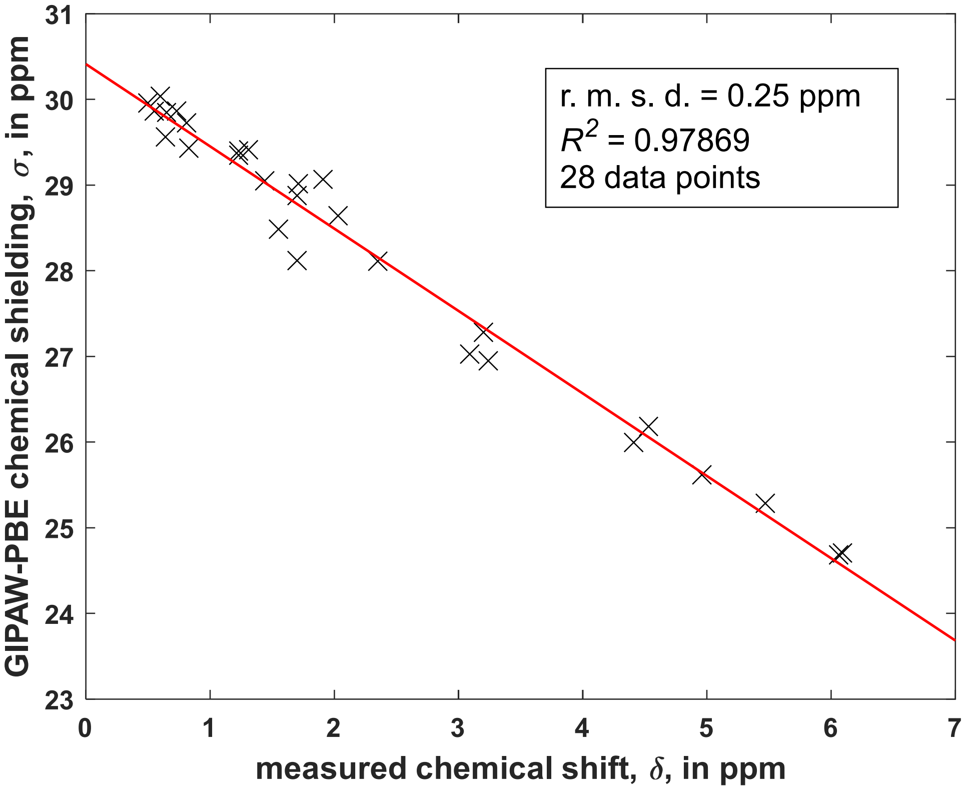

Figure S3. ^{1}H-^{13}C FSLG HETCOR NMR spectra of the high-temperature Form I of simvastatin measured at 300 K and 100, and 900 μs of CP mixing period.

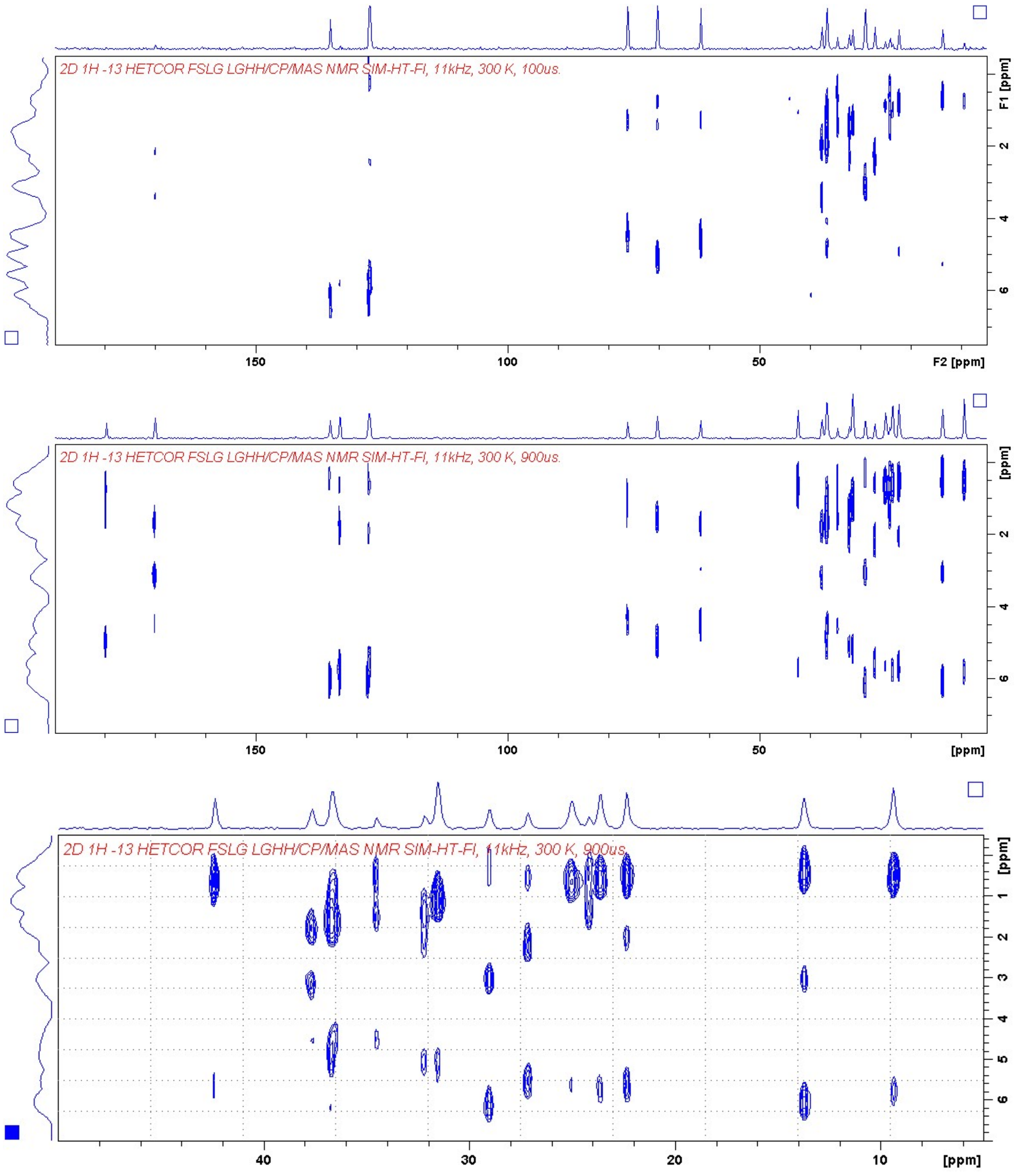

Figure S4. ^{1}H-^{13}C FSLG HETCOR NMR spectra of the low-temperature Form III of simvastatin measured at 225 K and 700 and 1600 μs of CP mixing period.

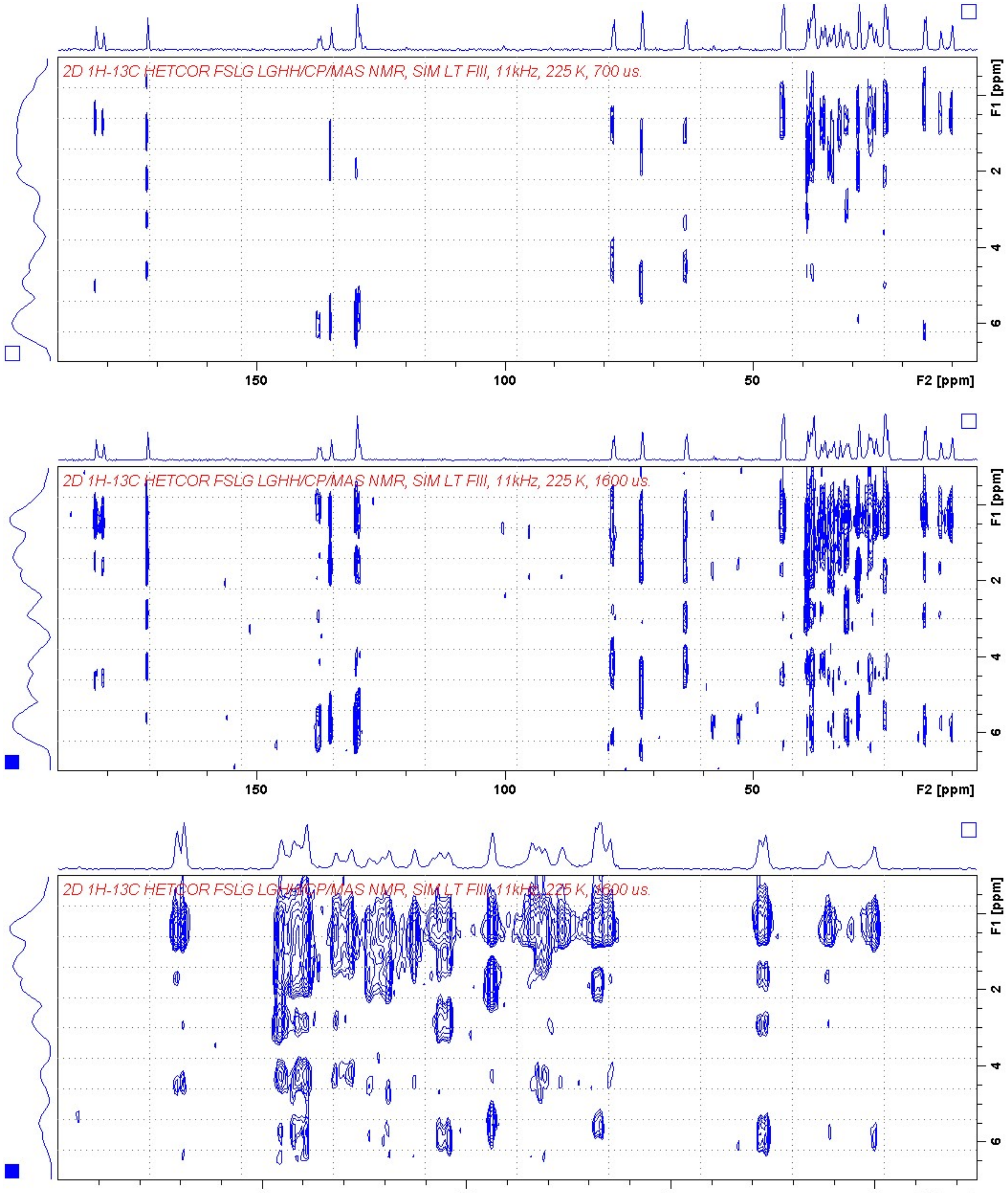

Figure S5. Full-range and expanded ^{1}H-^{13}C PILGRIM NMR spectra with the signal labeling of crystalline simvastatin measured at 310 K.

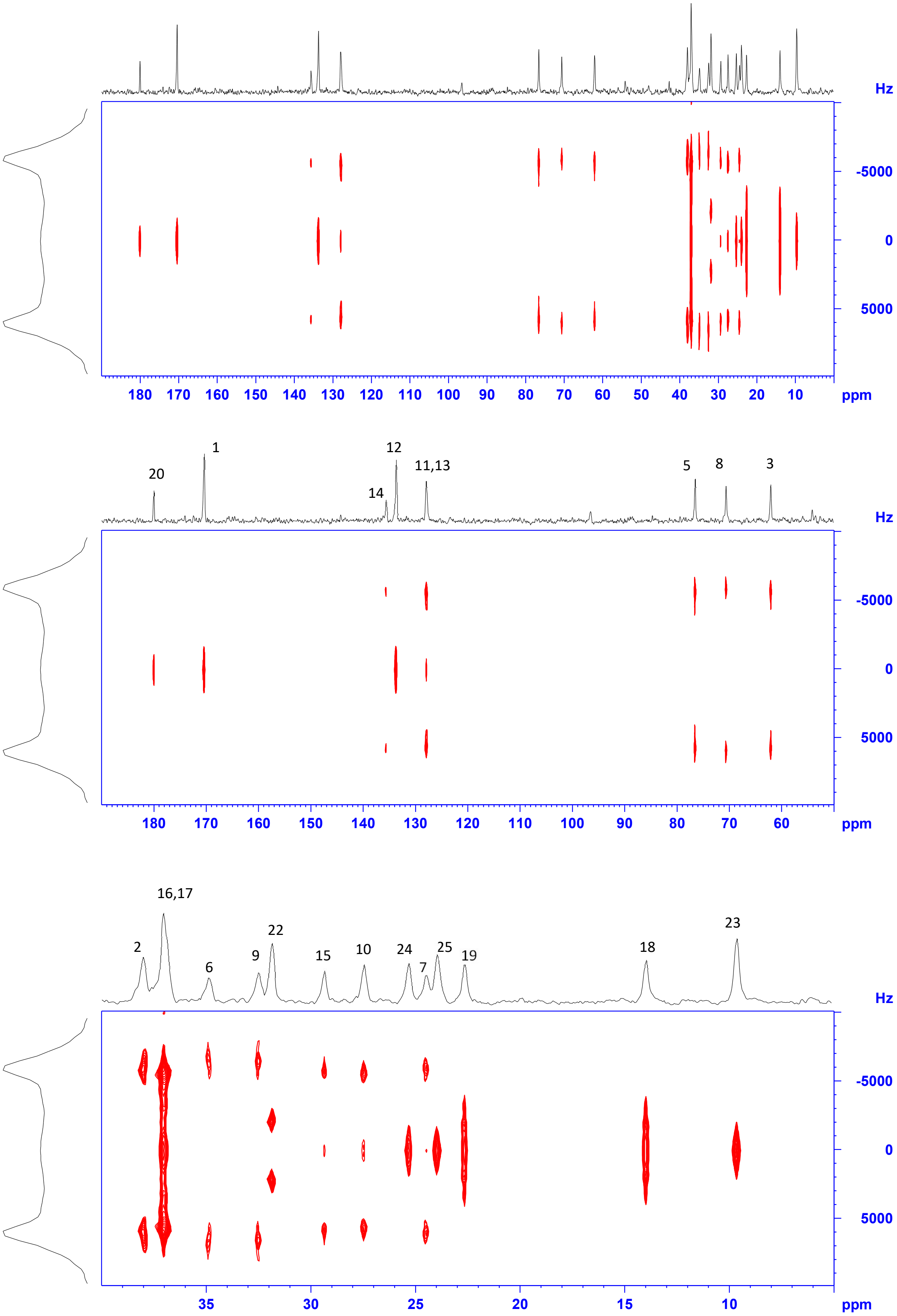

Figure S6. Full-range and expanded ^{1}H-^{13}C PILGRIM NMR spectra with the signal labeling of crystalline simvastatin measured at 257 K

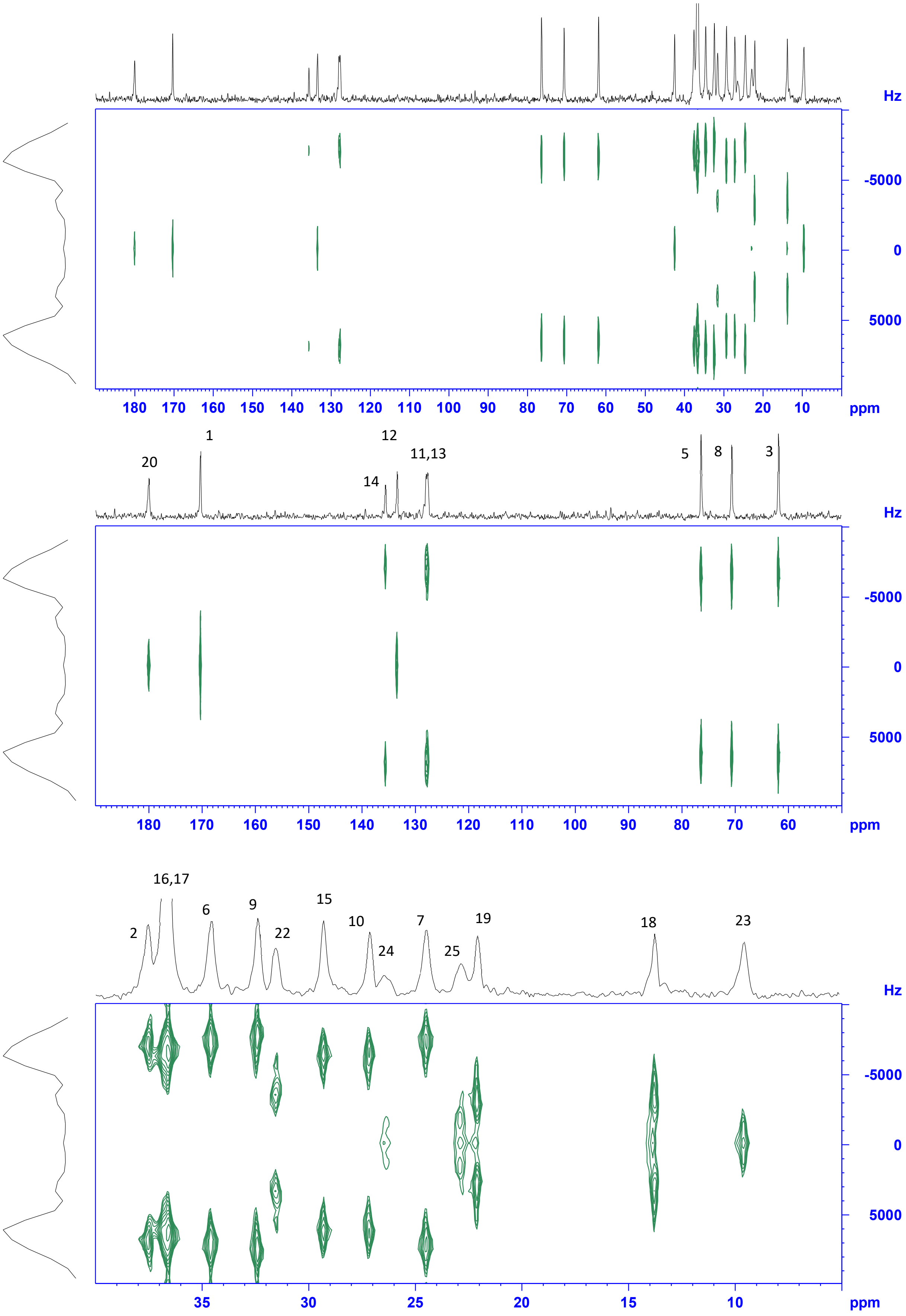

Figure S7. Full-range and expanded ^{1}H-^{13}C PILGRIM NMR spectra with the signal labeling of crystalline simvastatin measured at 231 K

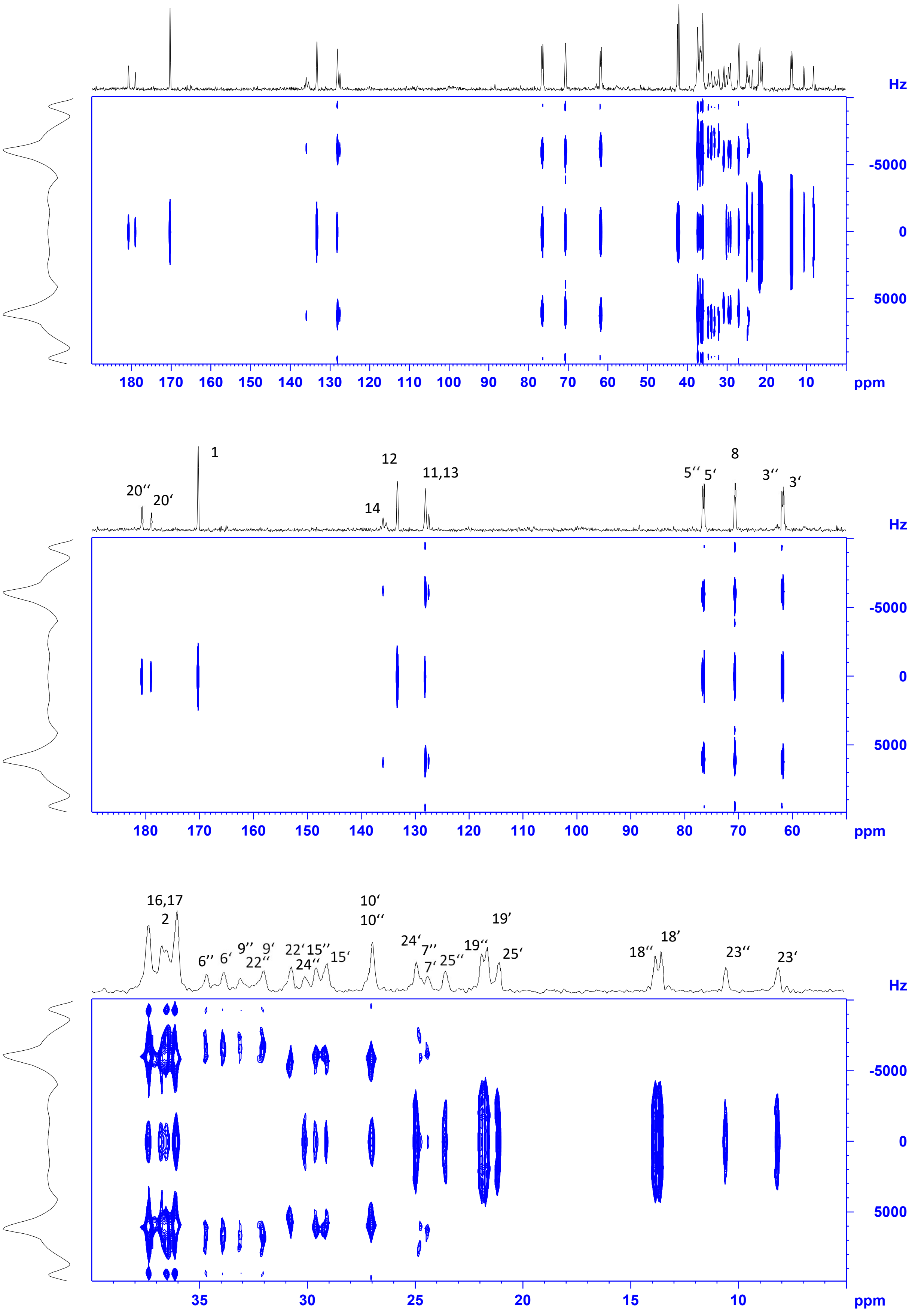

Supplement: Supplementary file 1 [file molecules-27-00679-s001.zip › SM-molecules-1513443-revised-for-publication-proof-read.pdf]
